# The Requirement for Pre-TCR during Thymic Differentiation Enforces a Developmental Pause That Is Essential for V-DJβ Rearrangement

**DOI:** 10.1371/journal.pone.0020639

**Published:** 2011-06-03

**Authors:** Karen S. Hathcock, Lila Farrington, Irina Ivanova, Ferenc Livak, Roza Selimyan, Ranjan Sen, Joy Williams, Xuguang Tai, Richard J. Hodes

**Affiliations:** 1 Experimental Immunology Branch, National Cancer Institute, National Institutes of Health, Bethesda, Maryland, United States of America; 2 Laboratory of Cellular and Molecular Biology, National Institute on Aging, National Institutes of Health, Baltimore, Maryland, United States of America; 3 Department of Microbiology and Immunology, University of Maryland School of Medicine, Baltimore, Maryland, United States of America; University of California, San Francisco, United States of America

## Abstract

T cell development occurs in the thymus and is critically dependent on productive TCRβ rearrangement and pre-TCR expression in DN3 cells. The requirement for pre-TCR expression results in the arrest of thymocytes at the DN3 stage (β checkpoint), which is uniquely permissive for V-DJβ recombination; only cells expressing pre-TCR survive and develop beyond the DN3 stage. In addition, the requirement for TCRβ rearrangement and pre-TCR expression enforces suppression of TCRβ rearrangement on a second allele, allelic exclusion, thus ensuring that each T cell expresses only a single TCRβ product. However, it is not known whether pre-TCR expression is essential for allelic exclusion or alternatively if allelic exclusion is enforced by developmental changes that can occur in the absence of pre-TCR. We asked if thymocytes that were differentiated without pre-TCR expression, and therefore without pause at the β checkpoint, would suppress all V-DJβ rearrangement. We previously reported that premature CD28 signaling in murine CD4^−^CD8^−^ (DN) thymocytes supports differentiation of CD4^+^CD8^+^ (DP) cells in the absence of pre-TCR expression. The present study uses this model to define requirements for TCRβ rearrangement and allelic exclusion. We demonstrate that if cells exit the DN3 developmental stage before TCRβ rearrangement occurs, V-DJβ rearrangement never occurs, even in DP cells that are permissive for D-Jβ and TCRα rearrangement. These results demonstrate that pre-TCR expression is not essential for thymic differentiation to DP cells or for V-DJβ suppression. However, the requirement for pre-TCR signals and the exclusion of alternative stimuli such as CD28 enforce a developmental “pause” in early DN3 cells that is essential for productive TCRβ rearrangement to occur.

## Introduction

In order to generate mature αβ T cells, developing thymocytes must negotiate developmental checkpoints that depend upon the rearrangement of TCR α and β genes. During conventional thymic development DN3 cells that express a surface pre-TCR consisting of a productively rearranged TCRβ chain, an invariant pre-TCRα chain, and CD3 components, successfully negotiate the “β checkpoint” and differentiate. Although circumstances have been described that allow the generation of DN4 cells in the absence of expressed TCRβ (and pre-TCR) or TCRγδ [1], [2] successful differentiation of the vast majority of DN4 thymocytes in adult mice requires pre-TCR signals to allow rescue from apoptosis, extensive proliferation, and development to CD4^+^CD8^+^ DP thymocytes [3], [4]. In addition pre-TCR signals also inhibit V-DJβ rearrangement on a second chromosome (allelic exclusion or β suppression) thereby assuring that each developing T cell expresses only a single TCRβ chain [5], [6]. We have studied TCRβ rearrangement and allelic exclusion and asked if these events absolutely require pre-TCR expression or alternatively if they are regulated by developmental changes that can occur in the absence of pre-TCR expression.

We previously reported that expressing CD28 and B7-2 as transgenes (CD28/B7) beginning in early DN3 cells replaced pre-TCR function at the β checkpoint. Expression of CD28/B7 transgenes in normal mice allowed the development of substantial numbers of DP cells that lack TCRβ expression. Further, when CD28/B7 transgenes were expressed in pre-TCR-deficient CD3εKO or RAG2KO mice, DP cells were also efficiently generated in the complete absence of pre-TCR expression [7]. In the studies reported here, we have used this murine model to examine developmentally induced changes in early DN thymocytes that regulate TCRβ rearrangement and allelic exclusion under conditions in which differentiation is not linked to pre-TCR expression. We asked whether thymocytes that are driven to differentiate without pause at the β checkpoint would ever undergo V-DJβ rearrangement.

This current study demonstrates that, in contrast to D–Jβ rearrangement, the opportunity for V-DJβ rearrangement occurs in a very limited developmental window that is restricted to early thymic DN3 cells [8], [9]. Pre-TCR expression is not required to enforce β suppression, but rather inhibition of V-DJβ rearrangement is enforced by at least two distinct and developmentally-regulated mechanisms that do not require pre-TCR expression: inhibition of RAG2 expression and, as measured by H3K4 tri-methylation and germline transcription, selective suppression of accessibility of the 5′ Vβ region. These findings support a novel model of pre-TCR function, namely, that the requirement for pre-TCR signaling enforces an essential “pause” in development at the early thymic DN3 stage that is uniquely permissive for TCRβ rearrangement and subsequent TCRαβ expression.

## Materials and Methods

### Animals

C57BL/6 (B6) mice were obtained from Frederick Cancer Research Facility. B7-2 transgenic Line 7 (B7), CD28WT transgenic (CD28), CD3εKO, and RAG2KO mice were generated as previously described [7]. The B7-2 Line 7 transgenic mice express murine B7-2 under the control of the H-2Kb promoter and the Ig μ chain enhancer [10] and the CD28WT transgenic mice express murine CD28 under the control of the human CD2 promoter/enhancer [11]. RAG2-GFP reporter mice were generously provided by M. Nussenzweig (Rockefeller University, New York City, NY). The indicated CD28, B7, and RAG2-GFP transgenic mice were bred onto the B6, CD3εKO or RAG2KO backgrounds, as indicated at Bioqual (Rockville, MD). All mice were used at 2–4 months of age. All animal experiments were approved by both the Bioqual and the National Cancer Institute Animal Care and Use Committees and assigned protocol numbers 09-3447-79 (Bioqual) and EIB 079 (National Cancer Institute).

### Antibodies

CD4-PE, CD4-biotin, CD8-biotin, TCRβ-FITC (H57), CD44-APC, CD25-PE (PC61), B220-biotin, TCRγδ-biotin(GL3), Mac-1-biotin, NK1.1-biotin, GR-1-biotin, and CD28-biotin antibodies (mAbs) were purchased from BD Biosciences (San Diego, CA). CD8-Alexa Fluor 647 (Al647) mAb was purchased from Invitrogen (Carlsbad CA). Steptavidin-PE-Cy5 (Av-PE-Cy5) and the BrdU-FITC staining kit were purchased from BD Biosciences. Streptavidin-Alexa Fluor 594 (Av-Al594) was purchased from Molecular Probes (Eugene, OR). FcγII/FcγIII (24G2)- and B7-2-specific (GL1) mAbs were previously described [7].

### Cell staining and purification

Single cell suspensions were prepared and stained as previously described [7] and analyzed by FACScan or FACScaliber (Becton Dickinson, San Jose, CA) using either FlowJo (Tree Star, Inc., Ashland, OR) or CellQuest (BD Biosciences) software. To identify DN thymocyte subsets, lineage-positive cells were incubated with biotin-conjugated mAbs specific for CD4, CD8, H57, TCRγδ, B220, Mac-1, NK1.1, and GR-1 followed by staining with anti-CD25 and anti-CD44 mAb. After extensive washing, Av-Al594 was added. DN cells were identified by gating on lineage-negative cells and forward scatter (FSC).

For some experiments DN cells were enriched by simultaneous CD4- and CD8-specific magnetic bead depletion (Milteny Biotec, Auburn CA), resulting in >94% pure DN cells that contained <1% DP cells. In select experiments DN2/3, DN4 cells, or DP cells were purified by flow cytometry (>98% pure).

### *In vivo* incorporation of BrdU

Mice were injected intraperitoneally two times with 1 mg BrdU at 4 and 2 hours prior to sacrifice. Thymi were harvested and surface stained as described above, followed by fixation, permeabilization and intracellular staining with anti-BrdU-FITC according to the manufacturer's instructions (BD Biosciences).

### Detection of TCRβ and TCRα rearrangement

DN2/3, DN4, and DP thymocytes were purified by flow cytometry; DNA was prepared using the Wizard Genomic DNA Purification kit (Promega, Madison,WI) and serial dilutions of DNA were assayed by semi-quantitative PCRs specific for Dβ2-Jβ2, Vβ5-Jβ2 and Vβ8-Jβ2 and Vβ14-Jβ2 rearrangements. DNA was also assayed by semi-quantitative PCRs specific for Vα2-Jα50.1, Vα2-Jα26, Vα10-Jα50.1, or Vα10-Jα26 rearrangements, and with IgM-specific primers as a loading control. Primers used are listed in Table S1. All PCRs were modified from previously published protocols [12]–[14], used Amplitaq Gold (Applied Biosystems, Foster City, CA), and were performed at the following conditions: 10 min at 95°C, (45 s at 94°C, 90 s at 65°C (TCRβ) or 50 s at 60°C (TCRα) , 150 s at 72°C)×32 cycles, 10 min at 72°C.

### Real-time PCR for RAG2 expression

DN2/3, DN4 or DP cells were purified by flow cytometry, and total RNA was isolated using the RNeasy kit (Qiagen, Valencia CA) with on-column DNase treatment. 400 ng RNA was converted to cDNA using the SuperScript®III First-Strand Synthesis System (Invitrogen, Carlsbad CA). SYBR® Green-based real-time PCR was performed using buffers and primers specific for RAG2 and, as a loading control, β-actin. All primers and buffers were purchased from SABiosciences (Frederick, MD). All real-time PCR assays were performed using replicate samples and two different concentrations of DNA. RT^−^ samples were included as a negative control in all experiments.

### Chromatin Immunoprecipitation (ChIP) analysis

RAG2KO cells (whole thymus) or enriched DN cells from CD28/B7/RAG2KO mice were subjected to ChIP as previously described [15] with minor modifications. Immunoprecipitating antibodies were obtained from Millipore (Billerica, MA). Formaldehyde crosslinked and sonicated chromatin from 5×10^6^ cells was pre-cleared with 5 µg non-specific IgG and immunoprecipitated with 5 µg anti-H3K4Me3 mAb or an equal amount of non-specific IgG. The precipitated DNA was purified, quantified fluorometrically and analyzed by real-time PCR to amplify the indicated TCRβ regions. Primers are listed in Supplementary Table S2.

### Detection of TCRβ germline transcription

Total RNA was prepared from enriched DN cells using the RNeasy kit (Qiagen). RNA was converted to cDNA using the SuperScript®III First-Strand Synthesis System (Invitrogen) and assayed using semiquantitative PCRs and primers specific for germline transcription of Dβ(1 and 2), Jβ(1 and 2) or Vβ(4.1, 5.1, 8.2, and 14.1), and for β-actin as a loading control. Primers are listed in Table S3. All PCRs were modified from previously established protocols [16], [17], use Amplitaq Gold (AppliedBiosystems, Foster City, CA), and were performed at the following conditions: for Dβ and Jβ: 10 min at 95°C, (20 s at 94°C, 30 s at 65°C, 60 s at 72°C)×35 cycles, 10 min at 72°C; for Vβ4.1 and Vββ14.1: 10 min at 95°C, (45 s at 94°C, 45 s at 65°C, 60 s at 72°C)×35 cycles, 10 min at 72°C; and for Vβ5.1 and Vβ5.1–8.2: 10 min at 95°C, (20 s at 94°C, 20 s at 54°C, 60 s at 72°C)×35 cycles, 10 min at 72°C. RT-minus samples were included as a negative control.

### CD3ε-induced differentiation of DP thymocytes in RAG2KO mice

As described previously [18], RAG2KO mice were injected IP with either PBS or 200 µg of CD3ε-specific mAb (2C11). Thymi were harvested at 3–6 days post-injection and analyzed by flow cytometry for CD4 and CD8 subsets. Enriched DN cells were prepared from individual mice by simultaneous CD4- and CD8-specific magnetic bead depletion.

## Results

### Expression of transgenic CD28 and B7-2 rescues development of DP thymocytes in the absence of pre-TCR

**Figure 1:**
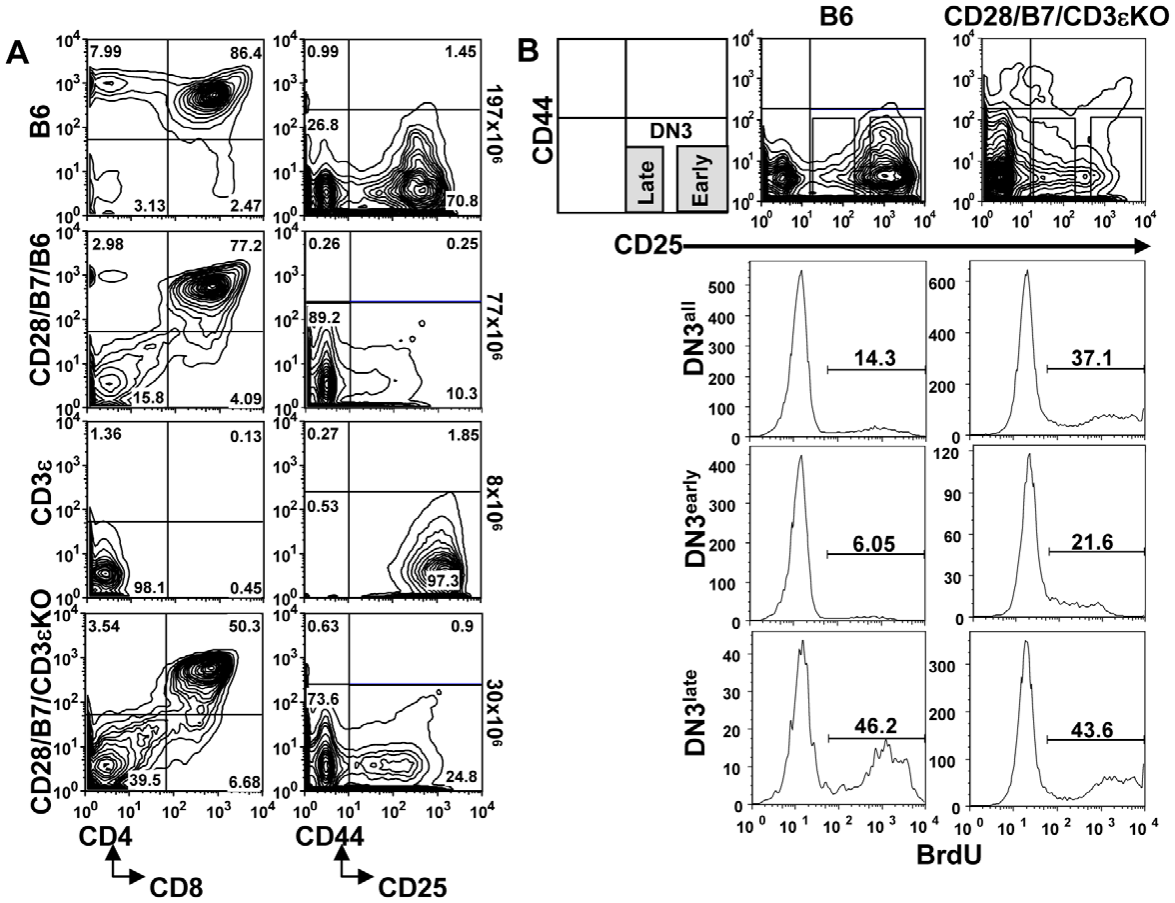
Analysis of thymic development and BrdU incorporation in mice undergoing pre-TCR-dependent or CD28/B7-dependent development. (A) Thymocytes from B6, CD28/B7/B6, CD3εKO, and CD28/B7/CD3εKO mice were analyzed for CD4-CD8 subsets (CD4-PE and CD8-Al594) or for lineage negative DN subsets (CD25-PE and CD44-APC). Thymus numbers are to the right of these figures. Staining profiles are representative of >40 mice of each genotype analyzed. (B) Thymi were harvested from mice injected with BrdU, lineage negative cells were gated on the indicated DN subsets, and analyzed for BrdU incorporation. These histograms show the percentage BrdU^+^ cells present in all DN3 cells, early DN3 (CD25^hi^) cells, or late DN3 (CD25^low^) cells from B6 or CD28/B7/CD3εKO mice. This experiment is representative of three independent experiments performed.

We previously reported that during conventional thymic development, CD28-B7 interactions prior to pre-TCR expression are limited and that expression of both CD28 and B7-2 as transgenes (CD28/B7) beginning early in DN cells substantially altered thymic development, bypassing the requirement for pre-TCR at the β checkpoint [7]. In wild-type B6 mice undergoing pre-TCR-dependent development, the DN3 to DN4 transition is rate limiting as cells rearrange and test for functional TCRβ; this developmental check point is reflected by a DN3 to DN4 ratio of 3.2+/−0.3 (n = 28). In contrast, in CD28/B7/B6 thymocytes the DN3 to DN4 ratio is substantially reduced (0.2+/−0.02, n = 15). In addition, expression of CD28/B7 transgenes on a CD3εKO background (CD28/B7/CD3εKO) reversed the developmental arrest observed in pre-TCR deficient CD3εKO mice and allowed the efficient generation of DP cells (Figure 1A) [8], [9]. The DN3 to DN4 ratio in CD28/B7/CD3εKO thymocytes (0.2+/−0.03, n = 9) is similar to that for CD28/B7/B6. The reversal of the DN3 to DN4 ratios observed in mice expressing transgenic CD28/B7 suggests that early CD28 signaling both replaces the requirement for pre-TCR function and accelerates development through the β checkpoint.

Further support for an accelerated development phenotype in the CD28/B7/B6 mice derives from experiments in which short-term labeling with BrdU was used to compare cell cycle regulation in DN3 cells undergoing pre-TCR-dependent or CD28/B7-dependent development (Figure 1B). In WT B6 mice only a low proportion of early DN3 (CD25^high^) cells were BrdU^+^ and the percentage of BrdU^+^ cells substantially increased in late DN3 (CD25^low^) cells following pre-TCR expression. Compared to WT B6 mice, there were 2.5-fold more BrdU^+^ DN3 cells in pre-TCR deficient CD28/B7/CD3εKO mice. Importantly, this increase in BrdU incorporation in CD28/B7/CD3εKO DN3 cells was entirely due to the 3–4-fold higher proportion of BrdU^+^ cells in early, but not in late DN3 cells. Thus, CD28/B7-dependent development accelerates the onset of proliferation in DN3 cells, such that a significant proportion of early DN3 cell are proliferating, prior to the stage at which completion of TCRβ rearrangement and acquisition of pre-TCR occurs in normal WT B6 mice.

### Pre-TCR-independent differentiation inhibits TCRβ rearrangement in DN thymocytes

**Figure 2:**
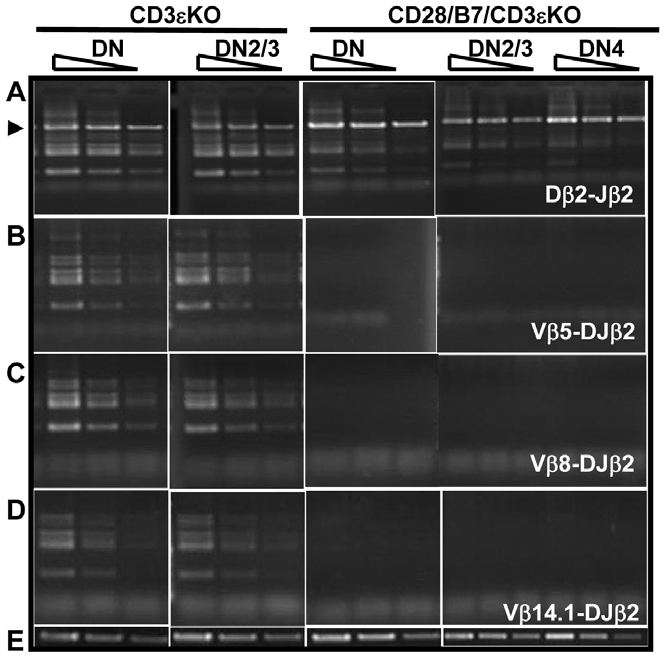
CD28/B7-driven development inhibits TCRβ rearrangement in DN thymocytes. 100, 30, and 10 ng of DNA was analyzed by PCR to detect (A) Dβ2-Jβ2 rearrangement, (B) Vβ5-DJβ2 rearrangement, (C) Vβ8.1-DJβ2 rearrangement, or (D) Vβb14.1-DJβ2 rearrangement. (E) 10, 3, and 1 ng of DNA was analyzed by PCR to detect unrearranged IgM as a loading control. The arrow in panel A denotes the amplification of germline or non-rearranged Dβ2-Jβ2 PCR products. These results presented here comparing VDJβ rearrangements in CD3ε and CD28/B7/CD3εKO DN cells were analyzed in the same experiment and are representative of three experiments analyzing DNA prepared from a total of six CD3εKO and CD28/B7/CD3εKO mice.

Expression of TCRβ protein occurs only after successful D-Jβ and V-DJβ rearrangement are completed [19]. Following TCRβ protein expression and pre-TCR formation, a series of differentiation events is initiated, and a critical component of this differentiation program is the suppression of additional V-DJβ rearrangement [6]. Since CD28/B7 interactions in early DN cells can induce differentiation in the absence of pre-TCR expression, we examined the effects of this differentiation program on TCRβ rearrangement and allelic exclusion. We began by analyzing TCRβ rearrangement in purified DN cells isolated from either pre-TCR deficient CD3εKO mice or from CD28/B7/CD3εKO mice. As previously reported [20], the absence of CD3ε precludes pre-TCR expression, and CD3εKO thymocytes, although arrested at the early DN3 stage of development, undergo extensive D-Jβ and V-DJβ rearrangement (Figure 2A). As compared to CD3εKO mice, Dβ2–Jβ2 rearrangement is substantially inhibited in all DN cells or in DN2/3 and DN4 subsets from CD28/B7/CD3εKO mice (Figure 2A). Further, analysis of Vβ5-, Vβ8-, and Vβ14.1-DJβ2 rearrangements in these same DN thymocytes reveal that V-DJβ rearrangement is undetectable in CD28/B7/CD3εKO DN cells (Figure 2 B–D). Thus, in the absence of pre-TCR expression the differentiation program induced by CD28/B7 expression in immature DN thymocytes results in at least a 10-fold inhibition of both Dβ2–Jβ2 and V–DJβ2 rearrangement in DN cells.

### Pre-TCR-independent development permits TCRα and D-Jβ but not V-DJβ rearrangement in DP thymocytes

**Figure 3:**
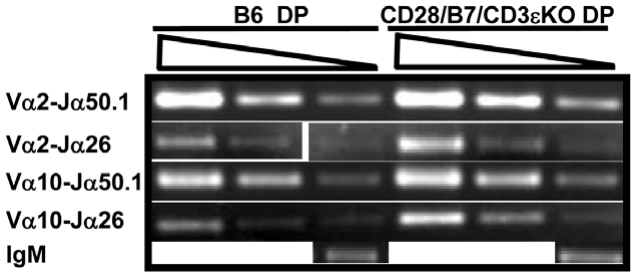
TCRα rearrangement is equivalent in DP cells generated via pre-TCR- or CD28/B7-dependent development. 300, 100, and 30 ng of DNA prepared from FCM sorted DP cells was analyzed by PCR to detect the indicated Vα-Jα rearrangements. The PCR reaction for unrearranged IgM was performed using 10 ng DNA. The results presented here comparing TCRα rearrangements in B6 and CD28/B7/CD3εKO DP cells were analyzed in the same experiment and are representative of three experiments performed using DNA prepared from five to six B6 or CD28/B7/CD3εKO mice.

During conventional thymic development, only DN cells that successfully rearrange TCRβ and express a pre-TCR can differentiate to DP cells and rearrange TCRα [3], [21]. We asked if CD28/B7-driven development would support TCRα rearrangement in DP cells that were generated from DN cells which failed to rearrange TCRβ and lack pre-TCR signaling. Despite the substantial inhibition of TCRβ rearrangement observed in DN cells from CD28/B7/CD3εKO mice (Figure 2), Vα-Jα rearrangement in DP cells from these same mice was not inhibited and, in fact, appeared comparable to that detected in DP cells isolated from WT B6 mice (Figure 3). This result demonstrates that CD28/B7-driven development is not universally inhibitory to TCR rearrangement and that efficient TCRα rearrangement in DP cells can occur in the absence of expressed TCRβ or pre-TCR signaling.

**Figure 4:**
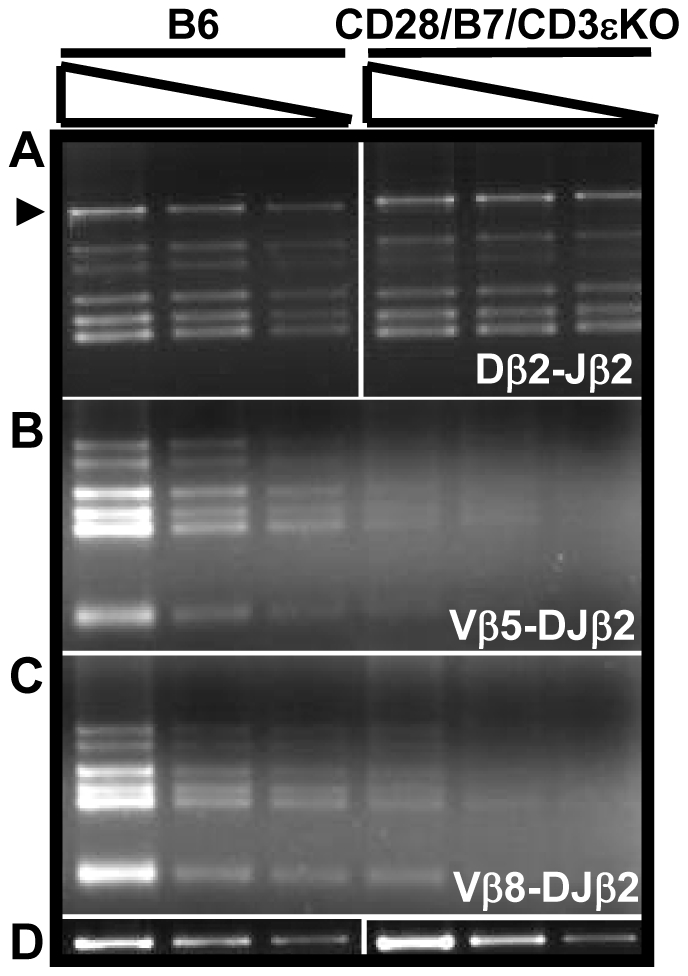
CD28/B7-dependent development permits Dβ2-Jβ2 rearrangement but substantially inhibits V-DJβ2 rearrangement in DP thymocytes. 100, 30, and 10 ng of DNA prepared from FCM sorted DP cells was analyzed by PCR to detect (A) Dβ2-Jβ2 rearrangements, (B) Vβ5-DJβ2, or (C) Vβ8.1-DJβ2 rearrangements. The PCR for unrearranged IgM, the loading control, was performed using 10, 3, and 1 ng of DNA (D). The results shown are representative of three experiments performed using DNA prepared from five to six B6 or CD28/B7/CD3eKO mice.

Since the recombination machinery necessary for TCRα rearrangement was intact in DP cells from CD28/B7/CD3εKO mice, we reexamined TCRβ rearrangement in these DP cells. In fact, Dβ2-Jβ2 rearrangement in DP cells from CD28/B7/CD3εKO mice was equivalent to that measured in pre-TCR-dependent DP cells from WT B6 mice (Figure 4A). However, in marked contrast, both Vβ5- and Vβ8-DJβ2 rearrangements were substantially inhibited in these same DP cells (Figure 4B–C). Since Dβ2 -Jβ2 rearrangement is deficient in DN thymocytes from CD28/B7/CD3εKO mice (Figure 2), this result demonstrates that during CD28/B7-induced development, D-Jβ2 rearrangement is delayed but occurs efficiently in DP cells. Importantly, however, even when D-Jβ rearrangement is successful, the developmental window in which V-DJβ rearrangement can occur is highly restricted. Suppression of V-DJβ rearrangement does not require pre-TCR expression but rather is developmentally enforced when DN cells exit DN3 and mature to DN4 and DP cells.

### CD28/B7-driven development inhibits RAG2 expression in DN but not DP cells

We next addressed the mechanism(s) that mediate suppression of V-DJβ rearrangement in the absence of pre-TCR. During conventional pre-TCR dependent development, availability of RAG proteins and accessibility of the TCRβ locus to recombination machinery play essential roles in regulating V(D)J recombination. Thus, pre-TCR expression correlates with the onset of extensive proliferation and other developmental changes that include inhibition of RAG expression and changes in TCRβ locus accessibility, both of which enforce allelic suppression [22]–[25]. However, it is unclear whether pre-TCR expression is absolutely required to induce these changes in RAG expression and locus accessibility or if developmental events unlinked to pre-TCR expression can also regulate these components. We therefore undertook a series of experiments designed to measure RAG2 transcription and TCRβ locus accessibility in DN and DP cells undergoing pre-TCR-dependent or CD28/B7-driven pre-TCR-independent development.

**Figure 5:**
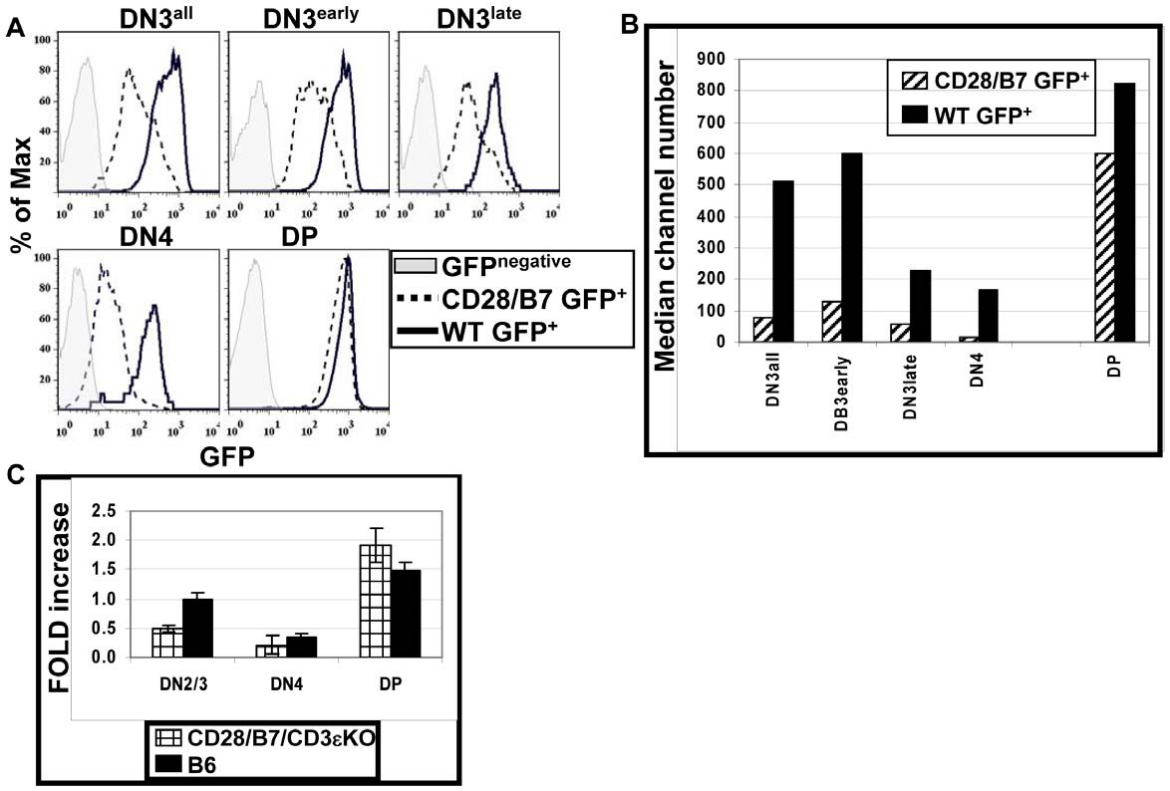
CD28/B7-dependent development suppresses RAG2 expression in DN but not DP cells. (A) Histograms show GFP^+^ (RAG2) staining on the FCM gated cell populations from WT GFP^+^ (solid line) or CD28/B7 GFP^+^ (dashed line) mice. Gates used to identify early and late DN3 subsets are as described in Figure 1B. The grey filled histograms show FL1 staining of the indicated DP and DN subsets analyzed from GFP^−^ mice. (B) This graph depicts the median channel number of GFP+ cells shown in panel (A) and is representative of three experiments performed. (C) This graph summarizes real-time PCR quantification of RAG2 expression in FCM-sorted DN2/3, DN4 and DP cells from B6 and CD28/B7/CD3εKO thymi. RNA was prepared and cDNA was synthesized as described in Materials and Methods. RAG2 cDNA was amplified using SABiosciences RT^2^qPCR primers. RAG2 expression was normalized against β-actin expression. Real-time analysis was performed on three independent cDNA samples for each genotype. Data are expressed as mean fold increase ± SE relative to B6 DN2/3 cells.

To study RAG2 expression we bred mice expressing both CD28 and B7-2 transgenes to a RAG2 transcriptional reporter mouse (RAG2-GFP) [26] and analyzed GFP expression in littermates that were RAG-GFP^+^ and either negative (WT) or positive for CD28/B7 transgenes (CD28/B7) (Figure 5A and B). In WT thymocytes undergoing pre-TCR dependent development, peak GFP expression is detected in DN3 cells, the stage in which TCRβ is actively recombining, and decreases as cells express a pre-TCR and differentiate to DN4 cells. GFP expression increases again as thymocytes differentiate to DP cells where TCRα undergoes recombination. Notably, GFP expression in DN3 and DN4 cells from CD28/B7 mice is substantially lower than that observed in comparable DN cells from WT littermates. Since TCRβ rearrangement normally begins in early DN3 cells, we evaluated GFP expression in the early and late DN3 subsets. As compared to GFP expression in early DN3 cells from WT littermates, GFP expression is significantly reduced beginning in early DN3 cells from CD28/B7 mice. GFP expression is comparably high in DP cells from both WT and CD28/B7 mice. These results demonstrate that the differentiation program induced by premature CD28/B7 expression inhibits RAG2 transcription beginning in early DN3 cells and that this inhibition continues throughout DN development. In contrast, CD28/B7-driven development does not prevent RAG2 re-expression in DP cells.

Similar results were obtained when real-time PCR was used to directly quantify RAG2 mRNA expression in sorted DN2/3, DN4, and DP cells isolated from either pre-TCR-dependent B6 and pre-TCR-deficient CD28/B7/CD3εKO mice (Figure 5C). In B6 DN thymocytes RAG2 expression peaks in DN2/3 cells and decreases in proliferating DN4 cells. As compared to RAG2 expression in DN2/3 cells from B6 mice, DN2/3 cells from CD28/B7/CD3εKO mice express approximately 50% less RAG2, and RAG2 expression is reduced further in DN4 cells. High expression of RAG2 is detected in DP cells from both B6 and CD28/B7/CD3εKO mice.

Thus, analysis of RAG2 by these two different experimental approaches reveals similar patterns of RAG2 expression and suggests that the accelerated developmental program initiated by CD28 signaling limits availability of RAG2 protein beginning in early DN3 cells. These results also demonstrate that the down regulation of RAG2 transcription that occurs as cells transit from DN3 to DN4 and the re-expression of RAG2 in DP cells are developmentally linked and do not require expression of a pre-TCR.

### CD28-driven pre-TCR-independent development selectively suppresses H3K4 tri-methylation of unrearranged Vβ genes

**Figure 6:**
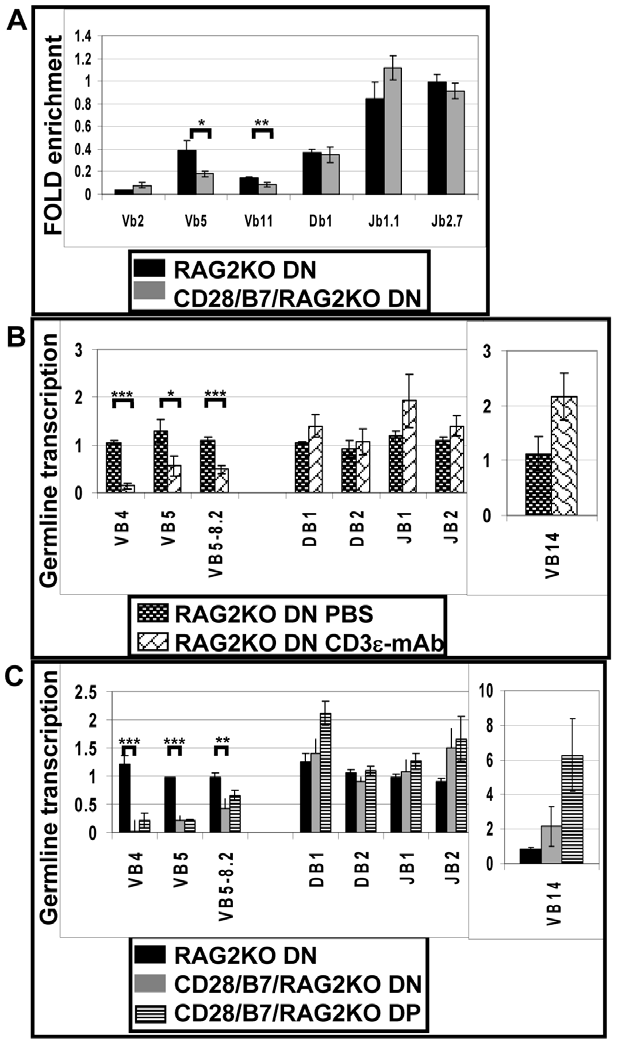
Accessibility of DJβ and Vβ regions are differentially regulated during thymic development. (A) Fold-enrichment H3K4Me3 analyzed by ChIP for Vβ (2, 5, and 11) Dβ1, and Jβ (1.1 and 2.7) regions. Each data point represents the mean ± SEM of six independent lysates prepared from RAG2KO (black bar) or CD28/B7/RAG2KO (grey bar) DN cells. H3K4Me3 of β-actin was used as a normalization control. (B) Relative germline transcription of the unrearranged TCRβ region in RAG2KO DN cells from mice previously injected with either PBS (dark brick bar) or CD3ε mAb (open brick bar). These results represent the mean ± SEM of three to five mice per group analyzed in two separate experiments. (C) Relative germline transcription of the unrearranged TCRβ region in RAG2KO DN cells (black bar), CD28/B7/RAG2KO DN cells (grey bar) or CD28/B7/RAG2KO DP cells (black striped bar). These results represent the mean ± SEM of five to six mice per genotype analyzed in two separate experiments. For both (B) and (C) data shown are normalized to transcription measured in RAG2KO DN cells using β-actin transcription as a loading control for each group. For (A), (B), and (C) the order of the data points reflects the 5′–3′ genomic organization of the TCRβ region and brackets identify comparisons that are significantly different (p<0.05): * indicates p≤0.03, ** indicates p≤0.02, and *** indicates p≤0.001.

We next asked if, in addition to suppressing RAG2 expression, development in the absence of pre-TCR expression also induces epigenetic modifications in the TCRβ locus that may inhibit accessibility of the locus to recombination or transcription machinery [27]. Tri-methylation of histone 3 at lysine 4 (H3K4Me3) is a physical modification that has been associated with RAG recruitment to accessible antigen receptor loci [28]–[30]. We used ChIP coupled to real-time PCR to compare H3K4Me3 across the entire unrearranged TCRβ locus in DN cells isolated from either RAG2KO mice or from CD28/B7/RAG2KO mice (Figure 6A) [7]. In DN cells from both RAG2KO and CD28/B7/RAG2KO mice, levels of H3K4Me3 were equivalently high in Dβ and Jβ regions. In contrast, analysis of Vβ loci indicated that H3K4Me3 modification of Vβ5 and Vβ11, but not Vβ2 regions was suppressed in CD28/B7/RAG2KO relative to RAG2KO DN cells. By this criterion the Dβ and Jβ but not Vβ regions of the TCRβ locus are available for recombination in CD28/B7 DN cells. These observations suggest that in addition to inhibition of RAG2 expression, impaired accessibility of Vβ gene segments also contributes to the inhibition of V-DJβ recombination in these developing DN cells.

### CD28/B7-driven or pre-TCR-driven development selectively suppresses germline transcription of unrearranged Vβ genes

We next assayed transcription of the unrearranged or germline TCRβ locus as a functional measure of chromatin accessibility [16], [17], [31]. Injection of RAG2KO mice with CD3ε-specific mAb is a well established in vivo model that mimics pre-TCR signaling and promotes the development of DP cells [18], [20], [32]. Analysis of TCRβ germline transcription in DN cells isolated from RAG2KO mice after injection with either PBS or CD3ε-specific mAb (Figure 6B) revealed that transcription of Dβ and Jβ regions was equivalently high in both treated and control groups. In contrast, after CD3ε mAb injection, germline transcription of Vβ genes, with the exception of the 3′ Vβ14, was substantially inhibited. The Vβ14 gene is unusual in that it is the only mouse Vβ gene located 3′ of the Dβ and Jβ regions, and it also has a transcriptional orientation that is opposite to that of the other Vβ genes. The selective inhibition of 5′Vβ, but not Dβ or Jβ, transcription reported here is similar to results previously published [20], [32]. These results suggest that pre-TCR-driven development enforces β suppression in DN cells by selectively inhibiting the accessibility of the 5′ Vβ locus to recombination machinery.

We next asked if the selective inhibition of Vβ germline transcription observed in this CD3ε-induced model of pre-TCR-driven development is unique or if inhibition of Vβ germline transcription also occurs if development is induced by transgenic CD28/B7 in the absence of pre-TCR expression (Figure 6C). Similar to the results described above for CD3ε-induced differentiation, Dβ and Jβ regions from RAG2KO DN cells and from CD28/B7/RAG2KO DN and DP cells expressed equivalent and high levels of germline transcription. Further, as compared to the robust transcription of the Dβ and Jβ regions, transcription of 5′ Vβ genes was selectively and substantially inhibited in both DN and DP cells from CD28/B7/RAG2KO mice. Thus, the selective inhibition of 5′Vβ but not Dβ or Jβ transcription also occurs in DN and DP cells that develop in the absence of pre-TCR expression. These results suggest that CD28/B7-driven development also enforces V-DJβ suppression in DN and DP cells by selectively inhibiting the accessibility of the 5′ Vβ locus to recombination machinery.

## Discussion

In the present study, we have used an experimental model of murine thymic development which disassociates the development of DP cells from the requirement for pre-TCR expression in order to identify requirements for TCRβ rearrangement and allelic exclusion. In this model, expression of transgenic CD28 and B7-2 beginning in early DN3 cells supports the efficient differentiation of DP thymocytes in the absence of pre-TCR expression [7]. This model reveals that pre-TCR expression is not required to enforce β suppression at the level of V-DJβ rearrangement or to allow TCRα rearrangement in DP cells. However, this differentiation pathway that occurs in the absence of a requirement for pre-TCR expression never allows productive TCRβ rearrangement to occur. Failure to rearrange D-Jβ in RAG-sufficient CD28/B7 DN cells (Figure 2A) is primarily the consequence of premature RAG2 suppression beginning in early DN3 cells since Dβ and Jβ regions remain accessible in both DN and DP cells. Consistent with this interpretation, D-Jβ rearrangement can occur in DP cells when RAG is re-expressed and is no longer limiting (Figure 4A). In addition to suppressing RAG2 transcription in DN cells, the developmental program induced by transgenic CD28/B7 expression in the absence of a requirement for pre-TCR also inhibits V-DJβ rearrangement in DN and DP cells by a second RAG-independent mechanism that results from the selective reduction in locus accessibility of the 5′ Vβ region (Figure 6). These results suggest that thymocyte development unlinked from a requirement for pre-TCR expression can nevertheless enforce β suppression not only by limiting RAG2 availability but also by selectively inhibiting the accessibility of the 5′ Vβ locus to recombination machinery.

**Figure 7:**
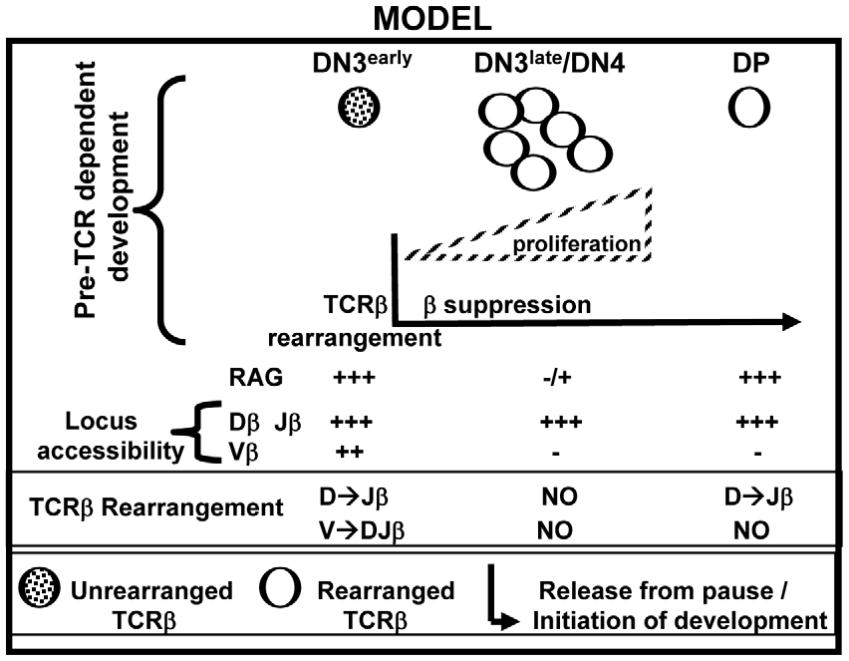
Model: The requirement for pre-TCR signaling enforces an essential pause in DN cell development.

These findings support a novel model for pre-TCR function in thymic development and V-DJβ recombination (Figure 7). During conventional pre-TCR-dependent differentiation TCRβ is rearranged in early DN3 cells, so that proliferation and concomitant down-regulation of both RAG expression and 5′ Vβ locus accessibility do not occur until after productive TCRβ rearrangement is complete and pre-TCR is expressed in late DN3 cells. Thus, early DN3 represents a developmental stage that is uniquely permissive for TCRβ rearrangement because of both high level RAG expression and TCRβ locus accessibility. If proliferation and differentiation are initiated in early DN3 cells prior to successful V-DJβ rearrangement, cells exit this uniquely permissive developmental stage and are not able to complete V-DJβ rearrangement during any subsequent differentiation stage. In contrast, the requirement for pre-TCR signaling to initiate differentiation of DN3 cells enforces an essential “pause” at this developmental stage which is uniquely permissive for V-DJβ rearrangement. This model emphasizes not only the importance of a pause at the DN3 stage that is permissive for TCRβ rearrangement, but also the necessity of preventing expression of other signaling molecules, such as CD28, which are capable of accelerating DN to DP differentiation, or of otherwise mimicking the effects of pre-TCR signaling, prior to V-DJβ rearrangement. The findings presented here are similar to previous reports demonstrating that transgenic TCR expression promotes development to DP cells and suppresses endogenous V-DJβ rearrangement [32]–[36]. The suppression of endogenous TCRβ rearrangement by TCR transgene in these systems may be mediated either by accelerating normal DN differentiation and/or by mimicking more specific pre-TCR signals that are required to enforce V-DJβ suppression.

During normal pre-TCR dependent development, TCRα rearrangement occurs subsequent to TCRβ rearrangement and pre-TCR signaling [3], [21]. We demonstrate that in CD28/B7/CD3εKO mice, TCRα rearrangement occurs efficiently in DP cells that are generated without rearranged TCRβ or pre-TCR expression. Expression of activated Lck [33], [37] or β-catenin [36] transgenes has previously been reported to bypass the β checkpoint and to rearrange TCRα in the absence of productive V-DJβ rearrangement. These results are consistent with results presented here and support a model in which TCRα rearrangement is developmentally regulated and independent of TCRβ rearrangement or unique pre-TCR signals.

During conventional thymic development high expression of RAG proteins is restricted to developmental stages in which TCRβ or TCRα rearrangement occurs [22]–[24]. While the signals that initiate RAG expression are not fully understood, it is known that levels of RAG proteins in developing cells are negatively regulated by proliferation, with both RAG2 transcription and protein stability inhibited in cycling cells [38]–[40]. In the present study, we report that CD28-driven differentiation accelerates the onset of proliferation in early DN3 cells and concomitantly suppresses RAG2 transcription beginning in early DN3 cells. Since it was reported that the cyclinA/CDK2-mediated phosphorylation of RAG2 in proliferating cells efficiently targets RAG2 for ubiquitin-mediated degradation [40], it is likely that the accelerated onset of proliferation induced by CD28 in DN cells targets RAG2 for degradation and limits RAG2 protein availability beginning in early DN3 cells when it is essential for TCRβ rearrangement. When differentiation and cell cycling is initiated by pre-TCR signals, RAG proteins are degraded only after productive TCRβ rearrangement is completed and pre-TCR is expressed. Thus, inhibition of RAG subsequent to pre-TCR expression prevents any additional V-DJβ rearrangement in DN cells and enforces β suppression. In contrast, if premature differentiation of DN cells is induced by alternative pathways, such as that mediated by CD28/B7, RAG expression is prematurely inhibited and productive TCRβ rearrangement can never occur, resulting in a sterile pathway that lacks TCRβ expression.

It is widely recognized that regulated RAG expression is not sufficient to completely explain the restricted patterns of V(D)J recombination [25], [38], [40] that are reported. The “accessibility hypothesis” first proposed by Alt and colleagues [27], states that V(D)J recombination is controlled by developmental and tissue-restricted chromatin modifications that differentially regulate accessibility of the TCR or Ig genes to recombination machinery. Selective suppression of Vβ region accessibility has been invoked to explain the fact that allelic expression is maintained in DP cells, where RAG is expressed and TCRα rearrangement occurs but V-DJβ rearrangement does not [6]. Previous reports, as well as the study presented here, have shown that selective suppression of Vβ but not Dβ or Jβ accessibility occurs when DN to DP development is induced by TCR transgenes [41] or CD3ε mAb injection [32]. However, it was not clear from these earlier studies whether down-regulation of Vβ region accessibility required expression of a pre-TCR. The observation that DP cells generated by transgenic CD28/B7 are permissive for V-Jα and D-Jβ rearrangement, but not for V-DJβ rearrangement suggests that developmental down-regulation of Vβ region accessibility in DP cells can occur independent of pre-TCR expression. To directly assess TCRβ accessibility, we first measured H3K4Me3 histone tri-methylation, a modification reported to be associated with actively transcribing genes and to bind to the RAG2 PHD domain [28]–[30], [42]. In the absence of pre-TCR expression, CD28-driven differentiation suppressed H3K4Me3 in some unrearranged Vβ loci, but not in Dβ or Jβ regions, suggesting that developmentally determined inhibition of 5′ Vβ region accessibility is a second independent mechanism mediating the suppression of V-DJβ rearrangement in developing DN cells. Germline transcription of non-rearranged TCR loci provides a functional measure of accessibility to transcriptional machinery, and has been widely identified as a prerequisite for rearrangement of T and B cell receptors [31]. Germline transcription of Dβ and Jβ loci was found to be equivalent in DN thymocytes from RAG2KO and CD28/B7/RAG2KO mice. In striking contrast, germline transcription of 5′ Vβ loci was markedly suppressed in DN thymocytes from RAG-deficient mice expressing CD28 and B7 transgenes. Germline transcription of TCRβ loci in DP cells induced by CD28 signals was similar to that detected in DN cells: suppressed transcription of 5′Vβ loci and robust transcription of Dβ and Jβ loci. These results demonstrate that, in contrast to Dβ and Jβ region accessibility, 5′ Vβ region accessibility is strictly confined to the DN3 stage of differentiation and that subsequent down-regulation of Vβ accessibility is not dependent on pre-TCR expression but rather is differentiation-induced.

The studies reported here demonstrate that the pre-TCR is not unique in its ability to drive thymocyte differentiation and to suppress V-DJβ rearrangement. A CD28-driven pre-TCR-independent pathway of development is sufficient to support differentiation while suppressing TCRβ rearrangement through effects on RAG2 expression and TCR Vβ locus accessibility. The requirement for pre-TCR expression to initiate differentiation, and the exclusion of alternative signaling molecules like CD28, ensure that development and suppression of V-DJβ rearrangement occurs only after productive TCRβ is complete.

## Supporting Information

Table S1 **PCR primers used to detect rearranged TCRβ and TCRα.**
(PDF)

Table S2 **PCR primers used to amplify TCRβ by ChiP.**
(PDF)

Table S3 **PCR primers used to detect germline transcription of unrearranged TCRβ.**
(PDF)
