# Supplementary material for: The Requirement for Pre-TCR during Thymic Differentiation Enforces a Developmental Pause That Is Essential for V-DJβ Rearrangement
Source: PLoS One. 2011 Jun 3;6(6):e20639. doi: 10.1371/journal.pone.0020639 (PMC3108609; doi:10.1371/journal.pone.0020639)
Supplement: Table S1 — PCR primers used to detect rearranged TCRβ and TCRα. (PDF) [file pone.0020639.s001.pdf]

Table S1. PCR primers used to detect rearranged TCRβ and TCRα.

PCR	Specificity	Primer	Reference
TCRβ	Dβ2-Jβ2	5'GTA GGC ACC TGT GGG GAA GAA ACT	(8,9)
		3' TGA GAG CTG TCT CCT ACT ATC GAT T	(8,9)
	Vβ2-Jβ2	5' CAG CAG ATT CTC AGT CCA ACA GTT T	(8,9)
		3' TGA GAG CTG TCT CCT ACT ATC GAT T	(8,9)
	Vβ8-Jβ2	5' TAT ATG TAC TGG TAT CGG CAG GAC A	(8,9)
		3' TGA GAG CTG TCT CCT ACT ATC GAT T	(8,9)
	Vβ14.1-Jβ2	5' AGA GTC GGT GGT GCA ACT GAA CCT	(8,9)
		3' TGA GAG CTG TCT CCT ACT ATC GAT T	(8,9)
TCRα	Vα2-Jα50.1	5' GCC GGA TCC AGG AGA AAC GTG ACC AGC A	(10)
		3' TCG TGG GAA AAT TGT AGG TTG T	(10)
	Vα2-Jα26	5' GCC GGA TCC AGG AGA AAC GTG ACC AGC A	(10)
		3' CCT GGA TCC TTA CTG TCA TAT ATC GAA	(10)
	Vα10-Jα50.1	5' AGC GAA TTC CCG CGT CCT TGG TTC TGC A	(10)
		3' TCG TGG GAA AAT TGT AGG TTG T	(10)
	Vα10-Jα26	5' AGC GAA TTC CCG CGT CCT TGG TTC TGC A	(10)
		3' CCT GGA TCC TTA CTG TCA TAT ATC GAA	(10)
Control	IgM[a]	5' CAC TAG CCA CAC CCT TAG CAC	(8)
		3' TGG CCA TGG GCT GCC TAG CCC GGG ACT T	(8)

[a]PCR for unrearranged IgM was used as a loading control for input DNA.
