# Supplementary material for: The Requirement for Pre-TCR during Thymic Differentiation Enforces a Developmental Pause That Is Essential for V-DJβ Rearrangement
Source: PLoS One. 2011 Jun 3;6(6):e20639. doi: 10.1371/journal.pone.0020639 (PMC3108609; doi:10.1371/journal.pone.0020639)
Supplement: Table S2 — PCR primers used to amplify TCRβ by ChiP. (PDF) [file pone.0020639.s002.pdf]

Table S2. PCR primers used to amplify TCRβ by ChiP.

Specificity	Primer	Reference
Vβ2	5’ GTC ACT GAT ACG GAG CTG AG	(11)
	3’ GTT TCA ATG CGC CTG CAG AG	(11)
Vβ5	5’ TGC CCA GCA GAT TCT CAG TC	(11)
	3’ GAG GGG AAG CGT ATG GTT TC	(11)
Vβ11	5’ CAA TCA GTC GCA CTC AAC TC	(11)
	3’ GAG GGG AAG CGT ATG GTT TC	(11)
Dβ1	5’ GAG GAA ACC CCT GCA TTA G	(11)
	3’ GGC TGG AAG AAA CCA CCA G	(11)
Jβ1.1	5’ ACT GGA GGG AAT CTA CCA TG	(11)
	3’ AAG AGA GGC TCT TCC CAG CTA G	(11)
Jβ2.7	5’ TTC ACA TCT CTC GCT TCC AC	(11)
	3’ CCC AGA AAG GGT GAA GTT G	(11)
β-Actin[a]	5’ GCC TTG CCT GTT CCT GCT C	(11)
	3’ CAG ACC ATA AAC TGT ATT TTT CTT ATT	(11)

[a]PCR for β-Actin was used as a normalization control for these experiments.
