# Supplementary material for: The Requirement for Pre-TCR during Thymic Differentiation Enforces a Developmental Pause That Is Essential for V-DJβ Rearrangement
Source: PLoS One. 2011 Jun 3;6(6):e20639. doi: 10.1371/journal.pone.0020639 (PMC3108609; doi:10.1371/journal.pone.0020639)
Supplement: Table S3 — PCR primers used to detect germline transcription of unrearranged TCRβ. (PDF) [file pone.0020639.s003.pdf]

Table S3. PCR primers used to detect germline transcription of unrearranged TCRβ.

Specificity	Primer	Reference
Dβ1-Cβ	5’ GTG AAT TCT TCC AGC CCT CAA G	Livak, unpublished
	3’ AGG GGA TCC CAC AGT CTG CTC GG	Livak, unpublished
Dβ2-Cβ	5’ TCT GGA TCC TGT CTC CCT GCC CCT G	Livak, unpublished
	3’ AGG GGA TCC CAC AGT CTG CTC GG	Livak, unpublished
Dβ2-Cβ	5’ TCT GAA TTC TAG CTG GGA AGA GCC TC	Livak, unpublished
	3’ AGG GGA TCC CAC AGT CTG CTC GG	Livak, unpublished
Jβ1-Cβ	5’ TCT GAA TTC TAG CTG GGA AGA GCC TC	Livak, unpublished
	3’ AGG GGA TCC CAC AGT CTG CTC GG	Livak, unpublished
Vβ4	5’ TGG GCT CCA TTT TCC TCA GTT G	(12)
	3’ GGC AGT CTG ATT GTC CAT AAG T	(12)
Vβ5	5’ TTG GGT TGC TCT CTT TCT	(13)
	3’ CCT CTC CAG TAC ATC ACC AG	(13)
Vβ5-8.2	5’ TTG GGT TGC TCT CTT TCT	(13)
	3’ CCA CAC ATC ACT GTG CAT CA[a]	(13)
	3’ CCC CAC ATC ACT GTG CAT CA[a]	(13)
Vβ14.1	5’ GCA GCT TCT CCG TGC TTA GGA T	(12)
	3’ CTT TCT CCT GGG CAT GTT CTT G	(12)
β-Actin[b]	5’ ATG CCA ACA CAG TGC TGT CTG GTG G	Hathcock, unpublished
	3’ CTG ATC CAC ATC TGC TGG AAG GTG	Hathcock, unpublished

[a]PCR for β-Actin was used as a loading control for input cDNA.
[b]PCR used an equal mixture of both primers.
